# Long‐term safety and efficacy of subcutaneous implantable cardioverter‐defibrillator compared with transvenous implantable cardioverter‐defibrillator in propensity score‐matched patients from Japan

**DOI:** 10.1002/joa3.70063

**Published:** 2025-04-11

**Authors:** Yuki Konno, Shingo Sasaki, Yuji Ishida, Yuichi Toyama, Kimitaka Nishizaki, Takahiko Kinjo, Taihei Itoh, Masaomi Kimura, Kazufumi Kato, Toshihiro Iwasaki, Hitoshi Umezaki, Shun Hirosawa, Hirofumi Tomita

**Affiliations:** ^1^ Department of Cardiology and Nephrology Hirosaki University Graduate School of Medicine Hirosaki Japan; ^2^ Department of Cardiac Remote Management System Hirosaki University Graduate School of Medicine Hirosaki Japan; ^3^ Department of Advanced Management of Cardiac Arrhythmias Hirosaki University Graduate School of Medicine Hirosaki Japan; ^4^ Department of Advanced Therapeutics for Cardiovascular Diseases Hirosaki University Graduate School of Medicine Hirosaki Japan

**Keywords:** defibrillation, inappropriate shock, Japanese, oversensing, subcutaneous implantable cardioverter‐defibrillator

## Abstract

**Background:**

Subcutaneous implantable cardioverter‐defibrillator (S‐ICD) has been reported to be non‐inferior to transvenous ICD (TV‐ICD) in terms of device‐related complications and inappropriate shock (IAS). We aimed to evaluate the long‐term clinical outcomes of S‐ICD compared with TV‐ICD in Japanese patients.

**Methods:**

We studied 315 consecutive patients (TV‐ICD, 167; S‐ICD, 148) who underwent ICD implantation. A propensity score matching analysis was performed to select patient subgroups for comparison (104 patients in each group). Clinical outcomes, including appropriate and inappropriate ICD therapy, procedure‐ and lead‐related complications, and mortality, were compared between the two groups.

**Results:**

During follow‐up (median, 1458 [interquartile range, 1353–1572] days), the cumulative incidence of appropriate shock therapy was 9.6% and 8.7% in the S‐ICD and TV‐ICD groups, respectively (*p* = 0.94). Although the S‐ICD group tended to have a higher IAS than the TV‐ICD group (5.8% vs. 1.9%), the difference was not significant (*p* = 0.19). Conversely, the cumulative incidence of procedural and lead‐related complications was significantly lower in the S‐ICD group (2.9% vs. 9.6%, *p* = 0.02). Notably, lead‐related complications were more common in the TV‐ICD group (*p* = 0.05). There was no difference in all‐cause mortality between the two groups (*p* = 0.75), and heart failure exacerbation was the most common cause of death in both groups.

**Conclusions:**

In propensity score‐matched Japanese patients with S‐ICD, the cumulative incidence of appropriate shock and mortality was comparable to those with TV‐ICD. There was no significant difference in the rate of IAS. Notably, patients with S‐ICD had fewer lead‐related complications than those with TV‐ICD.

## Graphical abstract

In propensity score‐matched Japanese patients with S‐ICD, the cumulative incidence of ICD shock therapies and mortality was comparable to those with TV‐ICD. Notably, patients with S‐ICD had fewer lead‐related complications than those with TV‐ICD.

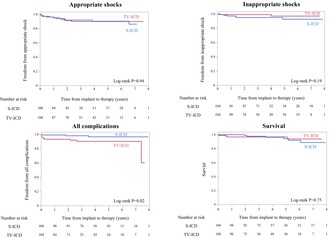

## 1 INTRODUCTION

The subcutaneous implantable cardioverter‐defibrillator (S‐ICD) is expected to be an alternative therapy to the transvenous ICD (TV‐ICD) for preventing sudden cardiac death (SCD). The PRAETORIAN randomized controlled trial recently demonstrated that S‐ICD is non‐inferior to TV‐ICD with respect to device‐related complications and inappropriate shock (IAS).^1^ Furthermore, the ATLAS study demonstrated that S‐ICD is associated with significantly fewer perioperative and lead‐related complications than TV‐ICD.^2^ Conversely, S‐ICD has been shown to result in higher rates of IAS than conventional TV‐ICD due to cardiac oversensing (OS), most commonly from T‐wave OS (TWOS).^3^, ^4^

The SMART Pass filter (SP; Boston Scientific Corporation, Natick, MA, USA) is a high‐pass filter that avoids cardiac OS below 9 Hz and is expected to be useful in the prevention of TWOS.^5^ Large‐scale Western studies have reported that the IAS rate with S‐ICD after the introduction of SP is comparable to that with TV‐ICD.^6^, ^7^ However, the SP often automatically deactivates due to sustained bradycardia or low QRS amplitude on the subcutaneous electrocardiogram (S‐ECG), and there remain issues to be addressed regarding its long‐term sustainability. Therefore, the long‐term safety and efficacy of S‐ICD with SP in Japanese patients remain unclear. Here, we investigated the long‐term safety and efficacy of S‐ICD with SP compared with TV‐ICD in propensity score‐matched Japanese patients.

## 2 METHODS

### 2.1 Study patients

This study included 315 consecutive patients who underwent ICD implantation at Hirosaki University Hospital between February 2016 and December 2023. Based on the Japanese Heart Rhythm Society guidelines, S‐ICD was selected over TV‐ICD for these patients who required an ICD but did not necessitate pacing therapy.^8^ Particularly, S‐ICDs were selected for patients with difficult venous access due to occlusion or removed TV‐ICDs due to infection, or for young patients who required long‐term ICD therapy as determined by their physician. As a result, 148 and 167 patients were implanted with S‐ICDs and TV‐ICDs, respectively.

Patient characteristics were analyzed from the baseline data, including age, sex, body mass index (BMI), underlying heart disease, the purpose of indication (primary or secondary prevention for SCD), left ventricular ejection fraction (LVEF), and blood test results (hemoglobin, serum creatinine, brain natriuretic peptide [BNP], or NT‐pro BNP levels). Procedure‐related complications were defined as hematoma, infection, or wound dehiscence requiring surgical intervention, and lead‐related complications were defined as lead fracture or lead dislodgement.

The institutional ethics committee approved the study protocol (2023‐178).

### 2.2 Propensity score matching analysis

To reduce the influence of the treatment selection bias and confounding factors, we used propensity score matching analysis. After comparing the clinical characteristics between the S‐ICD and TV‐ICD groups, significantly different variables between the two groups were used to create propensity‐matched pairs. Patients were matched on a 1:1 basis using the nearest neighbor method for significantly different variables. In the present study, 104 patients were selected for each group, as detailed in Section 3.

### 2.3 ICD programming and follow‐up

The choice of the TV‐ICD model and the detailed programming of each type of ICD was left to the physician's discretion. As a result, dual‐chamber ICDs were implanted in 95 patients and single‐chamber ICDs in 9 patients. Regarding manufacturer, BIOTRONIK (Berlin, Germany) ICDs were implanted in 46 patients, Medtronic (Minneapolis, MN, USA) ICDs in 43 patients, Boston Scientific (Marlborough, MA, USA) ICDs in 7 patients, and Abbott (Sylmar, CA, USA) ICDs in 8 patients. Therefore, several different discrimination algorithms were used, including SMART detection (BIOTRONIK), PR Logic and Wavelet (Medtronic), Rhythm ID (Boston Scientific), and Morphology Discrimination plus AV Rate Branch (Abbott). In patients with TV‐ICD, the ventricular fibrillation (VF) zone was ≥187 beats/min (bpm) with at least one train of anti‐tachycardia pacing (ATP) before the shock, and the ventricular tachycardia (VT) zone was ≥127 bpm, with at least three trains of ATP before the shock, which were allowed to be modified according to the patient's background. More detailed settings of the tachycardia detection rate by device manufacturers are shown in Tables S1 and S2. In patients with S‐ICD, the non‐conditional (shock) zone was >220–240 bpm, and the conditional zone was >170–190 bpm. SP was introduced immediately following S‐ICD implantation or at the time of device update in the outpatient clinic. If the SP was automatically deactivated, the analysis was censored at the point of deactivation to ensure consistency in the evaluation of the effect of the SP. Data collection on ICDs was performed by in‐person device interrogation during routine outpatient visits and remote monitoring.

### 2.4 Statistical analysis

Baseline characteristics were presented as medians (interquartile ranges [IQRs]). The Mann–Whitney *U* test was used for comparing continuous data. Fisher's exact test was used to compare the frequencies (percentages) of the categorical variables. For time‐to‐event outcomes, the Kaplan–Meier method was used for determining the cumulative incidence of outcomes, and the log‐rank test was used for comparing outcomes between groups. Data analyses were performed using JMP® 15 (SAS Institute Inc., Cary, NC, USA).

## 3 RESULTS

### 3.1 Baseline characteristics of the study patients

The baseline characteristics of all study patients are presented in Table 1. Of the 315 consecutive patients, 148 (47%) and 167 (53%) were implanted with S‐ICD and TV‐ICD, respectively. Comparisons of the baseline characteristics between the two groups showed that patients in the S‐ICD group were younger (median, 59 [IQR, 46–67] vs. 68 [IQR, 59–75] years, *p* < 0.0001), had higher serum creatinine levels (median, 0.95 [IQR, 0.76–1.25] vs. 0.92 [IQR, 0.78–1.14] mg/dL, *p* = 0.01), and a lower proportion of primary prevention (39.9% vs. 52.0%, *p* = 0.03) than those in the TV‐ICD group. There were no significant differences in sex, LVEF, and hemoglobin, BNP, or NT‐pro BNP levels between the two groups. The median BMI in the S‐ICD group was 24.1 [IQR, 21.1–26.9] kg/m^2^, and no difference was observed between the two groups.

**TABLE 1:** Baseline characteristics of the unmatched patients.

Clinical characteristics	S‐ICD group (*N* = 148)	TV‐ICD group (*N* = 167)	*p* value
Age, years	59 [46–67]	68 [59–75]	<0.0001
Male, *n* (%)	29 (19.5)	46 (27.5)	0.098
Body mass index, kg/m^2^	24.1 [21.1–26.9]	23.9 [21.5–26.9]	0.97
Left ventricular ejection fraction, %	48.3 [34.0–63.0]	45.0 [31–63.5]	0.98
Serum hemoglobin, g/dL	13.6 [11.6–14.9]	13.9 [12.4–14.8]	0.12
Serum creatinine, mg/dL	0.95 [0.76–1.25]	0.92 [0.78–1.14]	0.01
Primary prevention indication, *n* (%)	59 (39.9)	87 (52.0)	0.03
Diabetes, *n* (%)	52 (35.4)	54 (32.5)	0.60
Hypertension, *n* (%)	70 (47.6)	94 (56.6)	0.11
Dyslipidemia, *n* (%)	61 (41.5)	64 (38.6)	0.60
Hyperuricemia, *n* (%)	19 (13.0)	31 (18.7)	0.17

*Note*: Data are shown as median (IQR) or *n* (%). Three variables (age, serum creatinine, and primary prevention indication) show significant differences and were used to create propensity score‐matched pairs.

**FIGURE 1:**
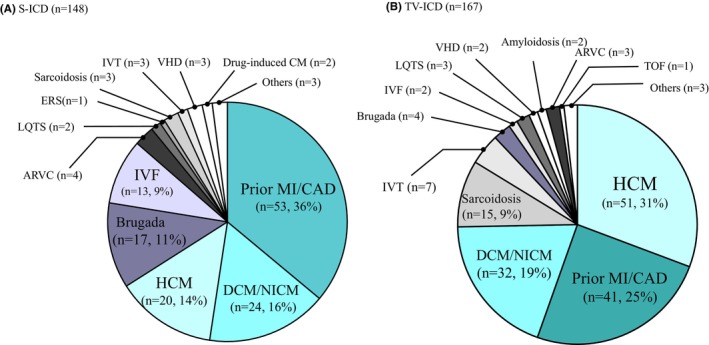
Underlying heart diseases in patients with (A) subcutaneous implantable cardioverter‐defibrillator (S‐ICD) and those with (B) transvenous implantable cardioverter defibrillator (TV‐ICD). ARVC, arrhythmogenic right ventricular cardiomyopathy; CAD, coronary artery disease; DCM, dilated cardiomyopathy; ERS, early repolarization syndrome; HCM, hypertrophic cardiomyopathy; IVF, idiopathic ventricular fibrillation; IVT, idiopathic ventricular tachycardia; LQTS, long QT syndrome; MI, myocardial infarction; NICM, non‐ischemic cardiomyopathy; TOF, tetralogy of Fallot; VHD, valvular heart disease.

In the S‐ICD group, the most common underlying heart disease was previous myocardial infarction (MI) or coronary artery disease (CAD) (*n* = 53, 36%), followed by dilated cardiomyopathy (DCM) or non‐ischemic cardiomyopathy (NICM) in 24 (16%), hypertrophic cardiomyopathy (HCM) in 20 (14%), Brugada syndrome (BrS) in 17 (11%), idiopathic VF (IVF) in 13 (9%), arrhythmogenic right ventricular cardiomyopathy (ARVC) in 4 (3%), cardiac sarcoidosis, idiopathic VT, valvular heart disease (VHD), and others (Figure 1A). In contrast, in the TV‐ICD group, the most common underlying heart disease was HCM (*n* = 51, 31%), followed by previous MI or CAD (*n* = 41, 25%), DCM or NICM in 32 (19%), cardiac sarcoidosis in 15 (9%), idiopathic VT in 7 (4%), BrS in 4 (2%), ARVC in 3 (2%), long QT syndrome in 3 (2%), IVF in 2 (1%), VHD in 2 (1%), and others (Figure 1B). There were more cases of inherited heart disease in the S‐ICD group, including BrS and IVF, than in the TV‐ICD group.

### 3.2 Propensity score‐matched patients

**FIGURE 2:**
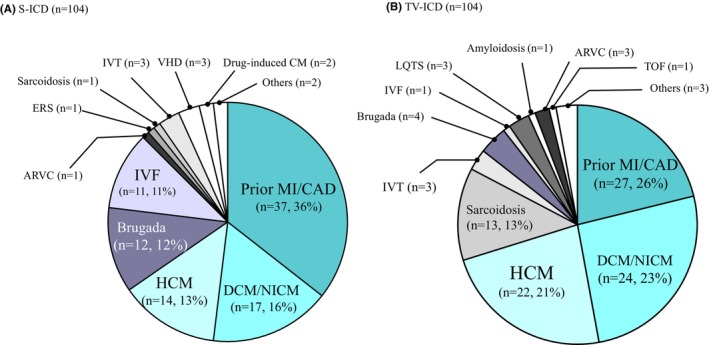
Underlying heart diseases in patients with (A) subcutaneous implantable cardioverter‐defibrillator (S‐ICD) and those with (B) transvenous implantable cardioverter defibrillator (TV‐ICD) after propensity score matching. ARVC, arrhythmogenic right ventricular cardiomyopathy; CAD, coronary artery disease; DCM, dilated cardiomyopathy; ERS, early repolarization syndrome; HCM, hypertrophic cardiomyopathy; IVF, idiopathic ventricular fibrillation; IVT, idiopathic ventricular tachycardia; LQTS, long QT syndrome; MI, myocardial infarction; NICM, non‐ischemic cardiomyopathy; TOF, tetralogy of Fallot; VHD, valvular heart disease.

Three variables that differed significantly between the S‐ICD and TV‐ICD groups (age, serum creatinine, and primary prevention indication) were used to create propensity score‐matched pairs, and 104 patients were selected for each group. The baseline characteristics of the propensity score‐matched patients were nearly identical in both groups (Table 2). As shown in Figure 2A,B, the most common underlying heart disease was prior MI or CAD in both groups, followed by DCM or NICM and HCM. Regarding the distribution of underlying heart disease, BrS and IVF were more common in the S‐ICD group, whereas sarcoidosis was more common in the TV‐ICD group.

**TABLE 2:** Baseline characteristics of the matched patients.

Clinical characteristics	S‐ICD group (*N* = 104)	TV‐ICD group (*N* = 104)	*p* value	SMD
Age, years	62 [56–69]	63 [54–75.75]	0.94	−0.01
Male, *n* (%)	23 (22.1)	30 (28.9)	0.26	0.15
Body mass index, kg/m^2^	24.5 [21.9–27.4]	23.8 [21.6–27.3]	0.93	0.01
Left ventricular ejection fraction, %	47.5 [34.0–63.0]	44.0 [30–57.5]	0.07	−0.25
Serum hemoglobin, g/dL	13.3 [12.9–13.7]	13.9 [12.5–14.8]	0.17	0.19
Serum creatinine, mg/dL	0.93 [0.75–1.21]	0.92 [0.78–1.28]	0.86	−0.02
Primary prevention indication, *n* (%)	42 (38.5)	43 (41.3)	0.89	−0.02
Diabetes, *n* (%)	40 (38.5)	35 (33.7)	0.47	−0.10
Hypertension, *n* (%)	51 (49.0)	52 (50.0)	0.89	−0.02
Dyslipidemia, *n* (%)	48 (46.2)	42 (40.4)	0.40	−0.17
Hyperuricemia, *n* (%)	14 (13.5)	20 (19.2)	0.26	0.16

*Note*: Data are shown as median (IQR) or *n* (%). Propensity scores for age, serum creatinine, and primary prevention indication were matched between the two groups. Standardized mean difference (SMD) represents the absolute difference between groups divided by the pooled standard deviation, with values <0.2 indicating adequate balance.

### 3.3 Incidence of appropriate ICD therapies in propensity score‐matched patients

**FIGURE 3:**
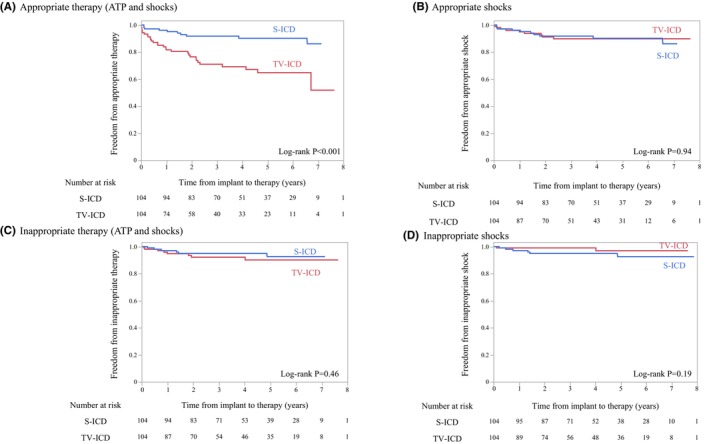
Kaplan–Meier plot of (A) appropriate therapy (ATP and shocks), (B) appropriate shocks, (C) inappropriate therapy (ATP and shocks), and (D) inappropriate shocks.

Over a median follow‐up period of 1458 (IQR, 1353–1572) days, the rates of all appropriate ICD therapies (ATP and shocks) were 9.6% (*n* = 10) and 28.8% (*n* = 30) in the propensity score‐matched S‐ICD and TV‐ICD groups, respectively. The TV‐ICD group had significantly higher rates of all appropriate ICD therapies than the S‐ICD group (log‐rank test, *p* < 0.001; Figure 3A). Conversely, in the mode‐specific analysis of appropriate ICD therapy, there was no significant difference in the incidence of shock therapy between the S‐ICD and TV‐ICD groups (9.6% vs. 8.7%, respectively; log‐rank test, *p* = 0.94; Figure 3B).

Of the 10 patients who experienced appropriate shocks in the S‐ICD group, 70% experienced sustained monomorphic VT, and 30% experienced VF. In contrast, in the TV‐ICD group, 97% of the spontaneous ventricular arrhythmias (VAs) were monomorphic VT, 67% of which were terminated with ATP alone. The mean rates of spontaneous VT were 236 ± 46 and 192 ± 38 bpm in the S‐ICD and TV‐ICD groups, respectively, indicating that the S‐ICD group had faster VT.

### 3.4 Incidence of inappropriate ICD therapies in propensity score‐matched patients

The SP was automatically deactivated in 7.7% (*n* = 8) of patients with S‐ICD. The two groups did not differ significantly in the rate of inappropriate therapy, whether all inappropriate therapy (S‐ICD group, 5.8% vs. TV‐ICD group, 7.7%; *p* = 0.46) (Figure 3C) or shock‐only inappropriate therapy (5.8% vs. 1.9%, respectively; *p* = 0.19) (Figure 3D). The IAS in the S‐ICD group was most frequently caused by TWOS due to several factors, including a temporal decline in the QRS amplitude of the S‐ECG and morphological changes in the S‐ECG due to intraventricular conduction disturbance (*n* = 4, 67%). The other causes of IAS in patients with S‐ICD were rapid atrial fibrillation (AF) (*n* = 1) and 2:1 atrial flutter (*n* = 1). Conversely, all inappropriate ICD therapies in the TV‐ICD group were caused by atrial tachyarrhythmias (*n* = 8, 7.7%). No patients developed IAS immediately after SP deactivation. Notably, 75% of the patients with inappropriate ICD therapies in the TV‐ICD group were treated with ATP alone and successfully avoided shock therapy.

### 3.5 Incidence of procedure‐ and lead‐related complications and mortality in propensity score‐matched patients

**FIGURE 4:**
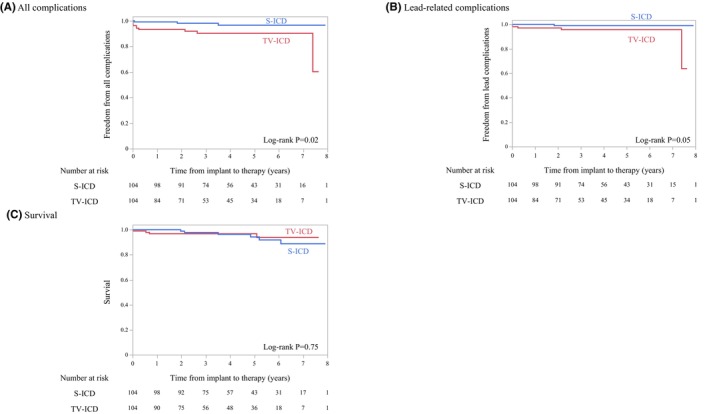
Kaplan–Meier plot of (A) all complications, (B) lead‐related complications, and (C) survival.

The S‐ICD group had a significantly lower cumulative incidence of procedure‐ and lead‐related complications during follow‐up than the TV‐ICD group (2.9% vs. 9.6%, *p* = 0.02) (Figure 4A). Notably, the S‐ICD group had fewer lead‐related complications than the TV‐ICD group (1.0% vs. 4.8%, *p* = 0.05) (Figure 4B).

During follow‐up (median, 1458 [IQR, 1353–1572] days), 6 (5.8%) and 4 (3.8%) patients in the S‐ICD and TV‐ICD groups died, respectively, and no significant difference in all‐cause mortality was observed between the two groups (*p* = 0.75) (Figure 4C). The most common cause of death in both groups was heart failure (S‐ICD group, 4; TV‐ICD group, 3), followed by non‐cardiac causes such as cerebral hemorrhage and infection.

## 4 DISCUSSION

### 4.1 Major findings

We here demonstrated the long‐term efficacy and safety of S‐ICD with SP compared with TV‐ICD in propensity score‐matched Japanese patients. The main findings of this study are as follows: First, patients in the TV‐ICD group received more appropriate ICD therapies due to the availability of ATP therapy; however, no significant difference in the rate of shock therapy was observed between the two groups. Second, in the S‐ICD group, the main cause of IAS was cardiac OS, especially TWOS, due to several factors, including decreased QRS amplitude on the S‐ECG over time and temporal changes in the QRS morphology; however, the SP reduced the number of IAS with TWOS alone. Consequently, there was no significant difference in the rate of inappropriate ICD therapy between the two groups. Finally, patients with S‐ICD had significantly fewer lead‐related complications than those with TV‐ICD.

### 4.2 Significance of ATP in ICD therapy

ATP is an effective treatment for terminating lethal VT without shock delivery in patients with TV‐ICD and is one of the useful ICD treatment strategies for preventing a decline in a patient's quality of life due to shock delivery.^9^ In this study, the TV‐ICD group had a significantly higher cumulative incidence of all ICD therapies than the S‐ICD group; however, the proportion of shock therapy was not significantly different. These results were probably due to the influence of tachycardia detection and treatment programming with TV‐ICD using ATP. In this study, although most patients in the TV‐ICD group were set to a long‐detection interval, which reduced the risk of overtreatment for spontaneously terminated VT, ICD therapy was performed significantly more often in the TV‐ICD group than in the S‐ICD group. One possible reason for this finding is that 60% of the patients in this study underwent implantation for secondary prevention of SCD, and the tachycardia detection rate was set to a lower rate that could detect clinical VT (Tables S1 and S2). In support of this hypothesis, most discrete VA events in the TV‐ICD group (*n* = 30, 97%) were ICD therapy for fast VT (mean VT rate, 192 ± 38 bpm); however, the VT rate was slower than that of the VT observed in the S‐ICD group (mean VT rate, 236 ± 46 bpm). Furthermore, 67% (*n* = 20) of all VTs detected in the TV‐ICD group (*n* = 30) were terminated with ATP, whereas 16% (*n* = 5) failed to terminate with ATP and ultimately required shock therapy.

In contrast, in the S‐ICD group, 70% (*n* = 7) of the discrete VA events were fast VT with a faster VT rate than in the TV‐ICD group, and all VA events were terminated with the first shock. In other words, the benefit of TV‐ICD is that it reduces the risk of shock delivery by using ATP; however, concerns have been raised about the risk of VT acceleration and the prolongation of the time required to terminate VT. Conversely, the benefit of S‐ICD is that it provides shock therapy without the need for ATP and can more quickly and reliably terminate VT.

### 4.3 Usefulness and limitations of the SP in preventing IAS in patients with S‐ICD

Patients with S‐ICD had a higher prevalence of IAS due to OS than those with TV‐ICD. In this study, 63% of IAS in the S‐ICD group resulted from OS. The S‐ECG used for sensing is a far‐field bipolar electrogram using two electrodes of the S‐ICD lead and a pulse generator, and three vectors (primary, secondary, and alternate) can be selected. As the S‐ECG is similar to the surface electrocardiogram, it is highly susceptible to the P and T waves and is highly sensitive to changes in the QRS/T ratio during exercise and to myoelectric interference due to certain movements. Although dual‐zone programming has significantly reduced IAS rates, the risk of IAS still remains regardless of the detection rate setting.^10^ The SP, recently introduced to S‐ICD, is a high‐pass filter that prevents the detection of low‐frequency signals below 9 Hz and is particularly useful in preventing TWOS. Moreover, in cases where the SP could be introduced, the IAS rate decreased to 4.3% in 1 year. However, the SP is often automatically deactivated due to sustained bradycardia or low QRS amplitude of the subcutaneous S‐ECG.^11^ In this study, the SP was automatically deactivated in 7.7% (*n* = 8) of patients with S‐ICD, indicating that there are still patients who cannot avoid IAS due to TWOS caused by a decrease in QRS amplitude over time or changes in QRS morphology.

In the S‐ICD group, 5.8% of patients experienced IASs despite the use of the SMART Pass filter, with approximately 70% of cases caused by cardiac OS related to progressive reductions in QRS amplitude or dynamic changes in QRS morphology. Furthermore, of the 8 patients with inappropriate ICD therapy in the TV‐ICD group, three had a history of AF and four were secondary prevention patients. Given that the tachycardia detection rate in TV‐ICD patients with an atrial arrhythmic substrate tends to be set lower to detect clinical VT, it is possible that more IAS due to atrial tachyarrhythmias occurred in these patients. These factors may explain why the incidence of IASs remained comparable between the two groups, even in the presence of the SP.

Conversely, the SP function remained activated in 92% of patients with S‐ICD, no OS was observed due to TWOS alone, and most of the causes of IAS were caused by TWOS due to several factors, including decreased QRS amplitude of the S‐ECG over time and changes in the S‐ECG configuration due to newly developed intraventricular conduction disorders. In most cases, preventing TWOS due to several factors by changing the sensing vector alone is challenging; in this study, lead repositioning was required in some cases. These results were limitations in preventing TWOS using SP alone; it was believed that selecting multiple sensing vectors at the time of implantation, selecting an optimal sensing vector that maintains the QRS amplitude of the S‐ECG for a long time, and setting a higher detection rate would be useful for IAS prevention.

Furthermore, although the SMART Pass filter is an important technological advance, the incidence of IAS remained comparable between the S‐ICD and TV‐ICD groups in the present study. These results suggest that the contribution of the SMART Pass filter to the prevention of TWOS requires further investigation. Therefore, the role of this algorithm should be interpreted with caution, and additional studies are warranted to validate its long‐term effects.

### 4.4 Risk of perioperative complications and selection of the optimal ICD device

It has been previously reported that S‐ICDs, in which the entire system is placed outside the blood vessel, have fewer lead‐related complications than TV‐ICDs due to the absence of mechanical stress on the lead caused by cardiac pulsation, the absence of chemical stress on the lead coating, and the expected long‐term durability of the lead structure without a stylet lumen. Additionally, the incidence of systemic infections is significantly lower than that of TV‐ICDs.^12^ Conversely, it remains unclear whether such benefits of S‐ICD can be extended to Japanese patients, who have a lower BMI than those in Western countries. In this study, consistent with the results of previous studies conducted in Western countries, the S‐ICD group had significantly fewer lead‐related complications and the incidence of wound‐related complications was similar to that of patients with TV‐ICD. It was inferred that the two‐incision technique, which requires fewer skin incisions, was used in most of the patients with S‐ICD and that an intermuscular pocket was created in all patients, which was effective in reducing the risk of complications even in Japanese patients with a low BMI. In cases where long‐term ICD use is required and pacing therapy is not indicated, the S‐ICD is considered an alternative therapy to the TV‐ICD. Although S‐ICD and TV‐ICD have fundamentally different lead placements, lead‐related complications remain a critical issue for device longevity and patient safety. The significantly lower incidence of lead‐related complications with S‐ICD underscores the benefits of avoiding transvenous access, particularly in patients at risk for venous occlusion or long‐term device complications.

### 4.5 Study limitations

This study has several limitations. First, its retrospective design may limit the generalizability of our findings. However, by studying consecutive patients admitted during the study period, we minimized the bias arising from the study design. Second, this study involved a limited number of patients in a single‐center setting. Third, the experience of the physician may have influenced the results because the selection and programming of each ICD type was at the physician's discretion. Fourth, patient follow‐up may have been too short to provide definitive evidence. Larger prospective studies are needed to validate our findings. Fifth, although propensity score matching was performed to balance baseline characteristics between the S‐ICD and TV‐ICD groups, there were differences in the distribution of underlying heart disease. In fact, the S‐ICD group included more patients with BrS and IVF than the TV‐ICD group, and the TV‐ICD group included more patients with sarcoidosis. These differences may still have influenced the clinical outcomes. Finally, the programming of TV‐ICDs, including high‐rate detection thresholds and prolonged duration settings, may have influenced the incidence of IAS. In addition, manufacturer‐specific algorithms, such as the iATP algorithm installed in the Medtronic ICD, may have contributed to differences in therapy delivery. Given the variability in device programming during the study period, establishing a standardized approach to ICD programming through expert consensus may further refine treatment outcomes.

## 5 CONCLUSIONS

In propensity score‐matched Japanese patients with S‐ICD, the cumulative incidence of appropriate shock and mortality was comparable to those with TV‐ICD. In addition, patients with S‐ICD had a similar cumulative incidence of IAS as those with TV‐ICD. Notably, patients with S‐ICD had significantly fewer lead‐related complications than those with TV‐ICD.

## FUNDING INFORMATION

This research was partly supported by the 3rd Sakurai Memorial Fund.

## CONFLICT OF INTEREST STATEMENT

Dr. Masaomi Kimura is an associate professor of the Department of Advanced Management of Cardiac Arrhythmias, which is an endowment Department supported by Medtronic Japan Co., Ltd., Japan Lifeline Co., Ltd., and Fukuda Denshi Kita‐Tohoku Hanbai Co., Ltd. Dr. Shingo Sasaki received a research grant from Boston Scientific Japan Co., Ltd. and is a concurrent associate professor of the Department of Advanced Management of Cardiac Arrhythmias and the Department of Cardiac Remote Management System, which is an endowment Department supported by BIOTRONIK Japan Co., Ltd. Dr. Hirofumi Tomita received a research grant from Abbott Medical Japan LLC. and is a concurrent professor of the Department of Advanced Management of Cardiac Arrhythmias, the Department of Cardiac Remote Management System, and the Department of Advanced Therapeutics for Cardiovascular Diseases, which is an endowment Department supported by Boston Scientific Japan Co. Other authors have no relevant disclosures.

## Supporting information


Data S1.
