# Supplementary material for: Long‐term safety and efficacy of subcutaneous implantable cardioverter‐defibrillator compared with transvenous implantable cardioverter‐defibrillator in propensity score‐matched patients from Japan
Source: J Arrhythm. 2025 Apr 11;41(2):e70063. doi: 10.1002/joa3.70063 (PMC11988202; doi:10.1002/joa3.70063)
Supplement: Supplementary file 1 — Data S1. [file JOA3-41-e70063-s001.docx]

**Supplementary Table 1.** Device manufacturer and tachycardia detection rate settings in patients implanted with TV-ICD for primary prevention

| **Manufacturer** | **VT rate** | **VF rate** |
| --- | --- | --- |
| BIOTRONIK (n=25) | ≥ 154〔150-174〕 | ≥ 200〔200-211〕 |
| Medtronic (n=12) | ≥ 178〔159-188〕 | ≥ 188〔188-188〕 |
| Boston Scientific (n=4) | ≥ 185〔140-200〕 | ≥ 200 (all patients) |
| Abbott (n=2) | ≥ 141〔140-141〕 | ≥ 200 (all patients) |

Data are shown as median (IQR).

**Supplementary Table 2.** Device manufacturer and tachycardia detection rate settings in patients implanted with TV-ICD for secondary prevention

| **Manufacturer** | **VT rate** | **VF rate** |
| --- | --- | --- |
| BIOTRONIK (n=21) | ≥ 150〔140-169〕 | ≥ 200〔200-227〕 |
| Medtronic (n=31) | ≥ 154〔143-188〕 | ≥ 188〔188-200〕 |
| Boston Scientific (n=3) | ≥ 223 (all patients) | ≥ 223 (all patients) |
| Abbott (n=6) | ≥ 161〔148-190〕 | ≥ 207〔197-214〕 |

Data are shown as median (IQR).
